# Design of Smart and Secured Healthcare Service Using Deep Learning with Modified SHA-256 Algorithm

**DOI:** 10.3390/healthcare10071275

**Published:** 2022-07-09

**Authors:** Mohan Debarchan Mohanty, Abhishek Das, Mihir Narayan Mohanty, Ayman Altameem, Soumya Ranjan Nayak, Abdul Khader Jilani Saudagar, Ramesh Chandra Poonia

**Affiliations:** 1 Department of Electrical Engineering, Campus 1, Technische Universität, 21073 Hamburg, Germany; mohan.debarchan97@gmail.com; 2 Department of Electronics and Communication Engineering, Institute of Technical Education and Research (ITER), Siksha ‘O’ Anusandhan (Deemed to be University), Bhubaneswar 701030, India; abhishekdas225@gmail.com; 3 Department of Computer Science and Engineering, College of Applied Studies and Community Services, King Saud University, Riyadh 11533, Saudi Arabia; aaltameem@ksu.edu.sa; 4 Amity School of Engineering and Technology, Amity University Uttar Pradesh, Noida 201303, India; nayak.soumya17@gmail.com; 5 Information Systems Department, Imam Mohammad Ibn Saud Islamic University (IMSIU), Riyadh 11432, Saudi Arabia; 6 Department of Computer Science, CHRIST (Deemed to be University), Bangalore 560029, India; rameshchandra.poonia@christuniversity.in

**Keywords:** hospital management system, deep learning, CNN-LSTM, DNN, encryption, SHA-256 algorithm

## Abstract

Background: The modern era of human society has seen the rise of a different variety of diseases. The mortality rate, therefore, increases without adequate care which consequently causes wealth loss. It has become a priority of humans to take care of health and wealth in a genuine way. Methods: In this article, the authors endeavored to design a hospital management system with secured data processing. The proposed approach consists of three different phases. In the first phase, a smart healthcare system is proposed for providing an effective health service, especially to patients with a brain tumor. An application is developed that is compatible with Android and Microsoft-based operating systems. Through this application, a patient can enter the system either in person or from a remote place. As a result, the patient data are secured with the hospital and the patient only. It consists of patient registration, diagnosis, pathology, admission, and an insurance service module. Secondly, deep-learning-based tumor detection from brain MRI and EEG signals is proposed. Lastly, a modified SHA-256 encryption algorithm is proposed for secured medical insurance data processing which will help detect the fraud happening in healthcare insurance services. Standard SHA-256 is an algorithm which is secured for short data. In this case, the security issue is enhanced with a long data encryption scheme. The algorithm is modified for the generation of a long key and its combination. This can be applicable for insurance data, and medical data for secured financial and disease-related data. Results: The deep-learning models provide highly accurate results that help in deciding whether the patient will be admitted or not. The details of the patient entered at the designed portal are encrypted in the form of a 256-bit hash value for secured data management.

## 1. Introduction

Due to the acceleration in information technology, the healthcare industries are adopting different novel approaches and models for smart healthcare. Smart systems are based on sensors, machine learning, artificial intelligence, and an advanced data communication approach. This smart and intelligent system allows the patients as well as the medical personnel, for an effective healthcare service. It includes patient registration, ambulance facility, diagnosis, causality management, pathology, patient admission, billing, and a health insurance management system [1]. Secured information processing in a smart healthcare system is one of the challenging tasks and it will be useful in controlling the fraud in medical sectors. Nowadays, fraud and abuse are the major issues in the healthcare sector. The major healthcare provider fraud happens in billing, drug, patient registration, insurance, ICU services, etc. [2]. Fraud in health insurance is increasing day by day and due to this, public-fund misutilization is also happening. Generally, in this type of fraud, the fake information is presented to the insurance company in an attempt to have them pay unofficial benefits to the policyholder, a different party, or the entity providing services. This type of fraud leads to severe losses for service providers as well as to the customers [3]. Health insurance fraud is mainly categorized into two types of (a) fraud related to consumers and (b) fraud related to a service provider [4,5]. Both cases happen due to the misuse and processing of information related to the insurance. The false claim, fake patient or insurance holder information with the wrong identity, and visiting of numerous physicians come under fraud related to the consumer whereas incorrect bills, fake prescriptions, pathology, and pharmacy-related fraud comes under service provider fraud. Due to this type of fraud, the medical industry is facing a huge amount of loss every year. To avoid such types of circumstances, a secured smart and intelligent healthcare management system is a priority.

Storing and processing the electronic health record (EHR) is one of the challenging tasks because it contains valuable information about the patient as well as the diagnosis process [6,7]. Accurate and secured processing of information related to the patient will help in accelerating the diagnosis process, improving healthcare, reducing the cost, and empowering the patients to effectively manage their health. Numerous technologies have been developed for presenting a highly secured healthcare data management system [8,9,10,11]. A deep-learning-based searchable encryption method has been proposed for secure data management [12]. The authors used blockchain technology along with a deep learning model for secure data searching by the users to avoid any misutilization of information. Blockchain technology has also been proposed in [13] for secure data encryption and scalability of the EHR system. The Ethereum protocol was found to be better in comparison to Dogecoin and Bitcoin protocols. Blockchain technology along with interplanetary file systems (IPFS) and attribute-based access control (ABAC) has been proposed for distributed HER systems [14]. The authors have utilized the break-glass concept in that work for real-time data access with high security. A patient-controlled HER system has been proposed using Ethereum protocol-based blockchain technology [15]. The authors have proposed that the healthcare provider would be able to access the patient data after obtaining consent from the patient. That method was designed to develop a patient-centric platform instead of a healthcare-provider-centric system. A blockchain data-storage-related problem has been addressed and its solution has also been proposed in [16]. The authors used hyperledger fabric to increase the storage of hashes of health records. For secured data management, an IPFS system was utilized. The application of blockchain technology in combination with heterogeneous signcryption with proxy re-encryption (HSC-PRE) has been proposed for secured data encryption and management [17]. Two main attributes of HER, interoperability and confidentiality of data, have been addressed in that work. A clinical HER system has been proposed to overcome the limitations in its usage [18]. Instead of using the HER system for administrative purposes only, the authors proposed a clinical data utilization for clinical usage using associated context parameters. A deep-learning-based approach has been adopted for IoT-enabled industrial EHR systems [19]. Blockchain technology was used as a distributed data storage for data privacy preservation with a keywords-based search for security. The features that are responsible for performance variation were studied and a cross-domain blockchain method was utilized for a secured EHR system to access the encrypted data [20]. A multiple-certificates authority including the patient was the main objective of that work. Application of machine-learning models has been found in [21] where the authors used the EMR system for data access, prophylaxis, and treatment. Various works have been proposed in the field of EHR. Still, there are some challenges in designing a highly tenable healthcare management system. The authors in this paper developed a three-stage smart and secured system to overcome the crisis in hospital management as well as patient service. The proposed approach is based on a three-stage structure. In the first stage, the advanced smart healthcare system is designed to perform the patient registration and diagnosis process. In the second stage, a deep-learning-based automatic disease classification system is adopted for classifying and diagnosing brain tumor patients. The last stage of the work is based on the secured health-insurance-data-processing system and for this, an encryption algorithm is considered to process the data related to the patient’s health insurance in an encrypted order.

The brain is the most essential part of living organisms. So, early diagnosis and immediate treatment of disease-related to the brain are highly required. Various methods have been developed to analyze brain images taken from different platforms such as computed tomography scans, magnetic resonance imaging, thermal imaging, etc. A few of such research developments in recent decades are considered in this section. A convolutional neural network (CNN) model developed with randomly generated graphs has been proposed in a recent work for classifying brain tumor MRI images [22]. The application of GeLU and ReLU activation functions in different layers of such a model is found. The random generation of graphs was performed using Erdos–Renyi (ER), Watts–Strogatz (WS), and Barabasi–Albert (BA) algorithms. Still, the test accuracy obtained in the modified model using these three algorithms was 95.49%, 95.17%, and 95.01%, respectively which needs to be improved further. A three-dimensional convolutional neural network has been used for brain image analysis for the diagnosis of acute brain hemorrhages [23]. The model is verified on a dataset consisting of around 12,000 CT images. As a pre-processing step, image thresholding has been applied to input images and fed to the CNN model. A transfer learning-based technique has been used for breast and brain histology image classification [24]. A pre-trained Google Inception V3 model was used in this method with simple modifications. The last four layers of this model were replaced by a global-average pooling layer, then four fully connected layers activated by ReLU, and finally a classification layer activated by a Softmax activation function. Ertosun and Rubin [25] used an ensemble model of two numbers of CNNs for the classification of different grades in brain glioma. Authors have classified the histopathology images of three types of glioma and obtained 96% accuracy for classification between glioblastoma multiforme (GBM) grade IV and lower-grade glioma (LGG), but a lower value of accuracy, i.e., 71% for classification of grade II and grade III types of glioma. The generative adversarial network (GAN) has been used in an application to enlarge the dataset on brain tumor MRI images [26]. The generated and original datasets were then fed to the CNN model for the classification of glioma sub-categories. 88.82% test accuracy was obtained with the hybrid model that needs improvement for a better diagnosis of brain tumors. The ensemble of machine-learning algorithms such as decision trees and bagging has been carried out for brain tumor classification [27]. The brain tumor histology slides were considered to contain tissues of oligodendroglioma. An ANN model was used to extract features and the classification was performed using the ensemble technique. The F-measure value obtained in this approach was 0.648 which is very little in contrast to the results obtained from deep-learning-technique-based models. In a recent study [28], it was observed that the applications of machine-learning techniques are also providing a competitive result in the era of deep-learning methods. The damaged-area evaluation has been proposed in [29] using the Gauss derivation theorem that provides a new direction in brain MRI processing. Parameter control-based optimization technique have been utilized for brain tumor detection using extreme gradient boosting ensemble model [30]. Ensemble learning-based models are gaining the attention. The application has been done in brain tumor detection [31]. A fuzzy min-max model was used in that work as the final classifier, stacked to the ensemble of CNN, recurrent neural network (RNN), long short-term memory (LSTM), and gated recurrent unit (GRU) models. Ensemble learning method has also been used for epileptic seizure detection from EEG signals [32]. In this work, we propose a transfer-learning-based approach with improved cross-entropy for better performance of disease diagnosis. A patient or the hospital management system needs secured data storage to avoid any consequences. Automatic diagnosis from the pathological reports and the decision to admit the patient is not yet focused on by state-of-the-art methods. A secured insurance module is also provided in this work to avoid any unwanted access and misutilization.

## 2. Smart Healthcare System

Nowadays, due to the advances in information technology, hospitals are adopting computer-aided services starting from patient admission to completing the diagnosis process. A smart and secured system is proposed in this work that will help to ensure effective hospital management. The total system consists of different modules and is described in the next subsections.

### 2.1. Login Module

**Figure 1:**
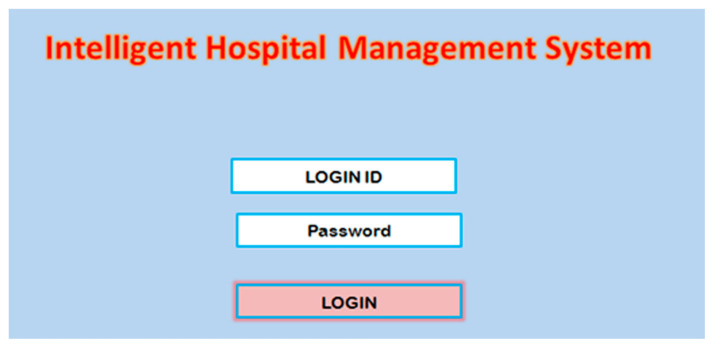
Login module.

The first module of the system is the login module. To start the service, the hospital staff first logs in by using the specific login ID and password. The diagram of the login module is presented in Figure 1. After successfully logging in to the application, the user is be able to access the different services such as patient registration, diagnosis, pathology, admission, etc.

### 2.2. Registration Module

**Figure 2:**
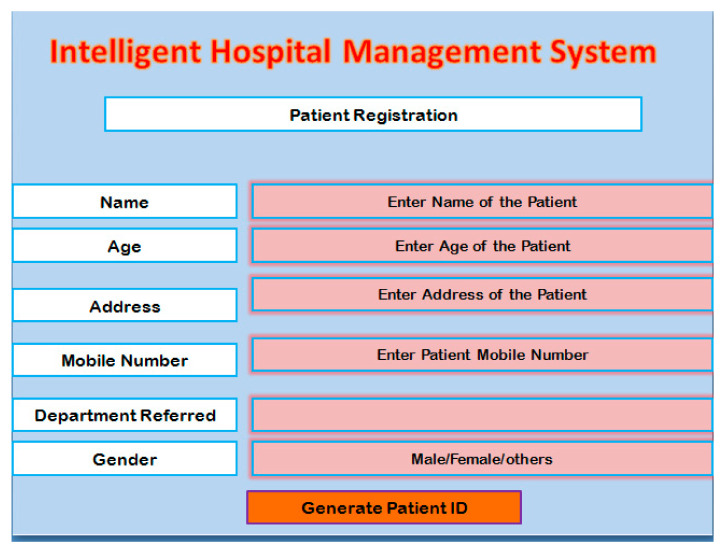
A patient registration module.

After the login step, the user is allowed to access different modules of the system. The first step is patient registration. Before starting the diagnosis process, the user performs the registration of the patient by providing different information as presented in Table 1. A screenshot of the registration module is presented in Figure 2. After providing the inputs to the system, it creates a unique patient ID. That ID is used for identifying the patient.

### 2.3. Patient Diagnosis Module

**Figure 3:**
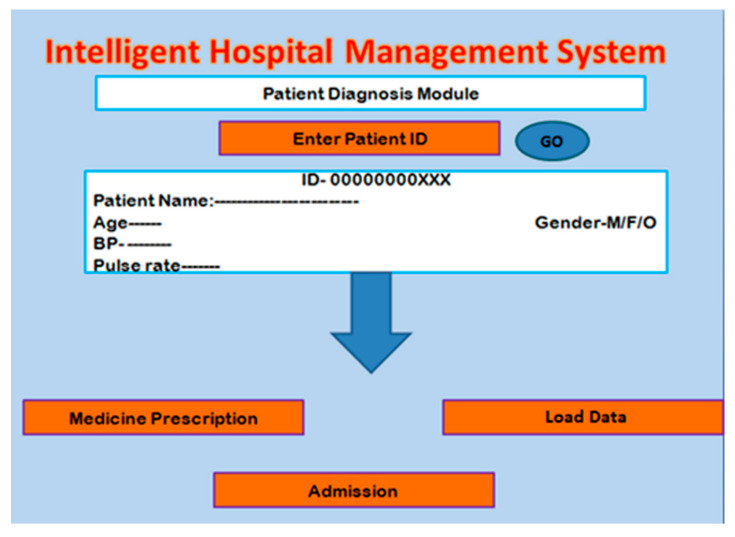
Patient Diagnosis Module.

After completion of the registration process, the patient is referred to the respective department for diagnosis. In the first step, the doctor enters the ID of the patient, and then the diagnosis process starts. Then, the electroencephalography (EEG) readings are taken along with brain magnetic resonance imaging (MRI). After completion of the diagnosis process, the doctor would attain the desired result and would prescribe the medicine with or without any pathological test. After the thorough analysis of the reports, the patient would either be advised for home medication or may be admitted to the hospital due to the severity. In case the doctor admits the brain tumor patient for diagnosis, in this work, we designed a diagnosis module for that scenario as shown in Figure 3.

Here, a deep-learning-based approach is proposed for MRI and EEG data classification. It will help for accelerating the patient diagnosis. A detailed description of the proposed deep-learning-based automatic classification model is described in the next subsections.

### 2.4. Deep-Learning-Based MRI and EEG Classification

**Figure 4:**
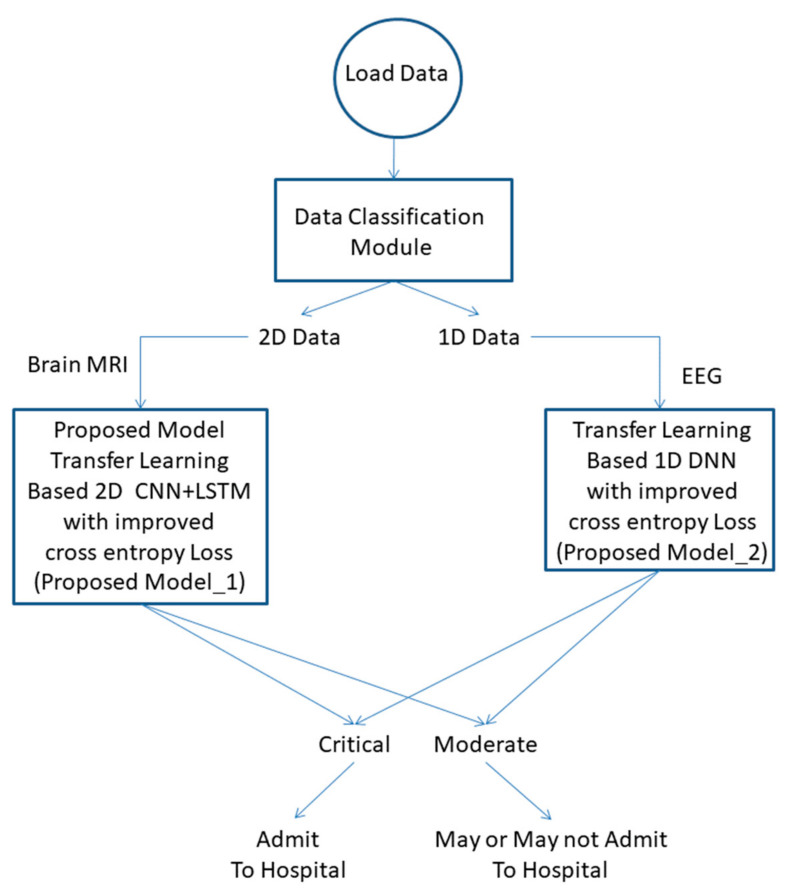
Block diagram of the proposed diagnosis model.

MRI is the most preferred method for the diagnosis of brain tumors. The presence of a tumor may lead to an instant seizure. A seizure can be monitored from EEG recordings. In this work, we propose a combined CNN-LSTM model for tumor detection from MRI and one-dimensional DNN for EEG classification. The proposed framework is shown in Figure 4.

#### 2.4.1. Data Classification Module

Data considered in this work are either two-dimensional (2-D) or one-dimensional (1-D). Brain MRIs are stored as 2-D data, whereas EEG readings are in 1-D. The size of the data is calculated using the size () function in the Python platform. The Algorithm 1 followed in this step is as follows:


**Algorithm 1: Data classification**
 Input: Data $\left(D\right)$ l represents the length of data Step 1. Evaluate the size of the data $\;\;size_{data}=size\left(D\right)$ Step 2.    If $size_{data}=h\;\times w\times depth$    then transfer D to the proposed mode_1 Step 3. If 
$size_{data}=1\;\times l$
   then transfer D to the proposed mode_2 Result: Data transfer to the corresponding model

#### 2.4.2. Transfer-Learning-Based CNN-LSTM Model

**Figure 5:**
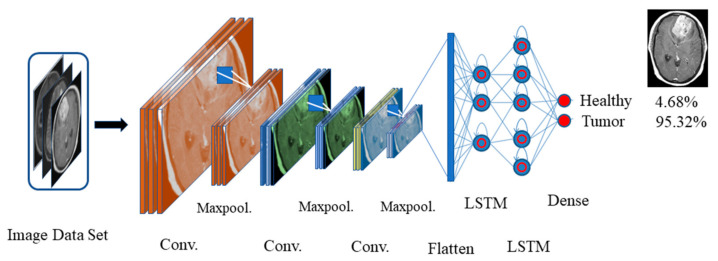
The proposed CNN-LSTM model.

We trained the 10-layered CNN model with a large-size dataset ImageNet so that we can transfer the learned weights for classifying small datasets for biomedical image classification. In this work, we replaced the classifier layer with two LSTM layers followed by a dense layer containing two nodes for healthy versus tumor classification trained with our small-size dataset. The newly added LSTM layers are trained with a brain tumor dataset [33]. The loss is calculated with modified cross-entropy. The proposed CNN-LSTM model is shown in Figure 5.

The models are evaluated using an improved cross-entropy loss as described in the next sub-section.

#### 2.4.3. Transfer-Learning-Based DNN Model

The EEG signals are classified for seizure detection. The 1-D DNN model is proposed with improved cross-entropy. The proposed model consists of one input layer, eight hidden layers and one output layer. Initially, the DNN model is trained with the University of Bonn (UoB) dataset [34] that contains 500 EEG data from five categories, i.e., F, N, O, S, and Z. The training algorithm follows the improved cross-entropy loss strategy to train the model. Transfer learning is applied by replacing the last three layers of DNN with three dense layers trained with the NSC-ND dataset [35] that contains a total of 150 EEG signals. The last layer of the new DNN now contains only three nodes to classify ictal, inter-ictal, and pre-ictal signals.

#### 2.4.4. Improved Cross-Entropy

The cross-entropy $\left(CE\right)$ was designed not only considering the predictions $\left(y^{\prime }\right)$ versus target $\left(y\right)$ data, but also we considered the total number of correct data $\left(C_{d}\right)$ as another parameter. The general CE is given by Equation (2) and the updated cross-entropy is mathematically represented in Equation (2).

(1) $$CE(y_{real}\;,\;y_{pred})=-\overset{2}{\underset{I=1}{\sum }}(y_{real}\log y_{pred})$$

(2) $$CE(y_{real}\;,\;y_{pred})=-\overset{2}{\underset{I=1}{\sum }}(y_{real}\log y_{pred})+\left(C_{d}-P_{d}\right)$$

where $P_{d}$ represents the number of data correctly predicted.

The cross-entropy loss is decreased until the result of Equation (2) becomes equal to that of Equation (1). This is only possible when the model is fully trained and $C_{d}=P_{d}$. This criterion is adopted for both the proposed models in this work.

The health condition of the patient is divided into two categories, i.e., critical and moderate, depending upon the EEG stability. The patient will be admitted to the hospital or will be released if decided accordingly.

### 2.5. Patient Admission Module

**Figure 6:**
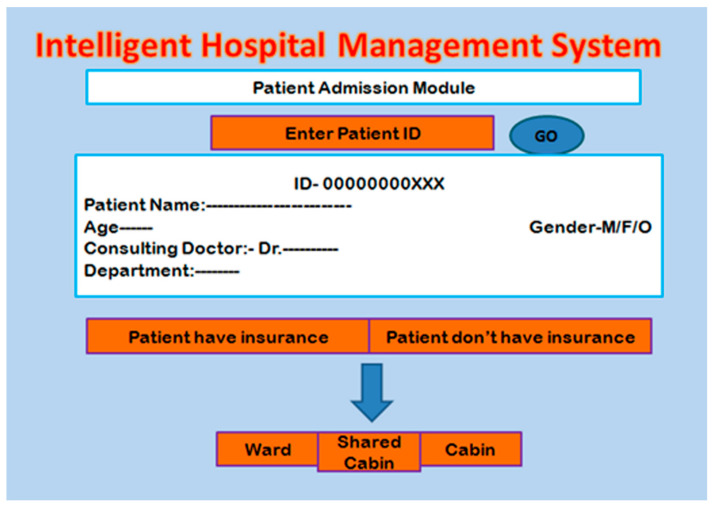
Patient admission module.

After the diagnosis process, the patient may be forwarded for admission for better treatment. The admission module checks the details of the patient by entering the unique patient ID generated at the time of registration; then, it shows the details such as disease, doctor name, and other pathological reports. The next step is to check whether the patient has health insurance or not. If the patient has insurance, then the bed is directly allocated after processing of the insurance, as presented in the next subsection, or else the direct bed allocation is performed. The bed preference is allocated depending upon the availability and patient preference. The screenshot of the patient registration module is shown in Figure 6.

### 2.6. Security Issue

The SHA-256 algorithm provides an output that contains 64 hexadecimal characters for 256 bits whereas the MD5 and MD4 algorithms contain 32 hexadecimal characters for 128 bits [36]. This makes it easier for any hacker to decode algorithms such as MD5. On the other hand, MD5 can be bypassed by generating collisions on various commercial computers, whereas SHA 256 is difficult as it is very long and there will be a greater number of combinations of collisions. This also makes SHA 256 slightly slower to handle than MD5. The RSA algorithm is the standard type that cannot be accommodated in this type of application [37].

#### 2.6.1. Insurance Module

**Figure 7:**
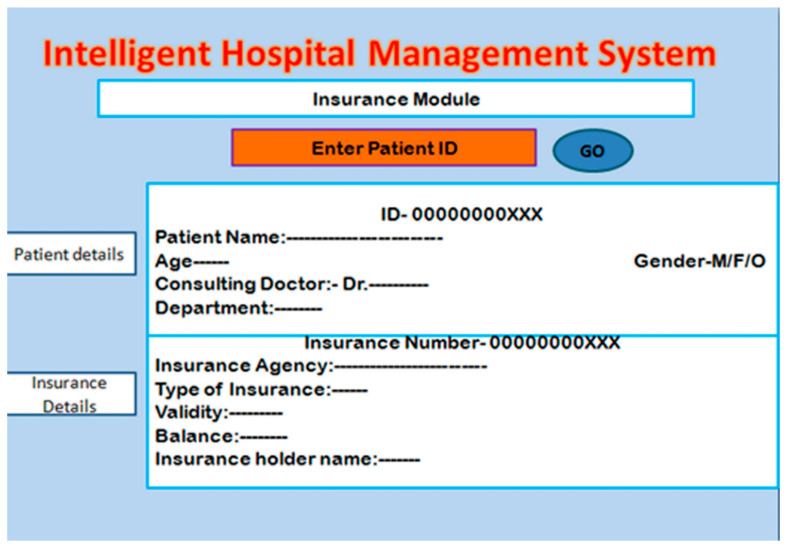
Patient insurance check module.

The insurance check module and processing the related information securely are the main objectives of this work. In this module, the user enters the patient and insurance ID of the patient to check the information related to the health insurance of the patient. A screenshot of this module is shown in Figure 7. After checking the details, the hospital authority starts the process by sending the approval of the insurance provider agency. To avoid fraud, the data need to be transferred in encrypted form. For this, a novel hashing-based encryption technique is proposed and described in the next subsection.

#### 2.6.2. Hashing the Medical Management Data for Security

Hashing is a mutation of a string of characters into a limited fixed-length key that addresses the initially entered string. The process is utilized in databases to store and recover data securely as it helps to access the data in the fastest possible manner with the help of digests or hash values. The hash values are preimage-resistive as they cannot be decrypted by any of the third-party users except the confidential user.

HL7 is a set of international standards used to transfer and share data between various healthcare providers. More specifically, HL7 helps bridge the gap between health IT applications and makes sharing healthcare data easier and more efficient. The implementation of SHA 256 for hashing would not affect the standards as the same data can be used after encryption by the other healthcare providers once the access is provided by the parent healthcare. In this paper, a modified cryptographic approach of SHA-256 is utilized for the security of a confidential medical database involving the insurance of a patient. SHA-256 is a part of the family of SHA-2-based hashing where a character string is mutated to a 256-bit digest. There are two novelties in this particular work. First is the modification of the compression function in the SHA-256 algorithm. Second is the appending of the data of a few textboxes into a single input text and hashing it to obtain a secured output.

**Figure 8:**
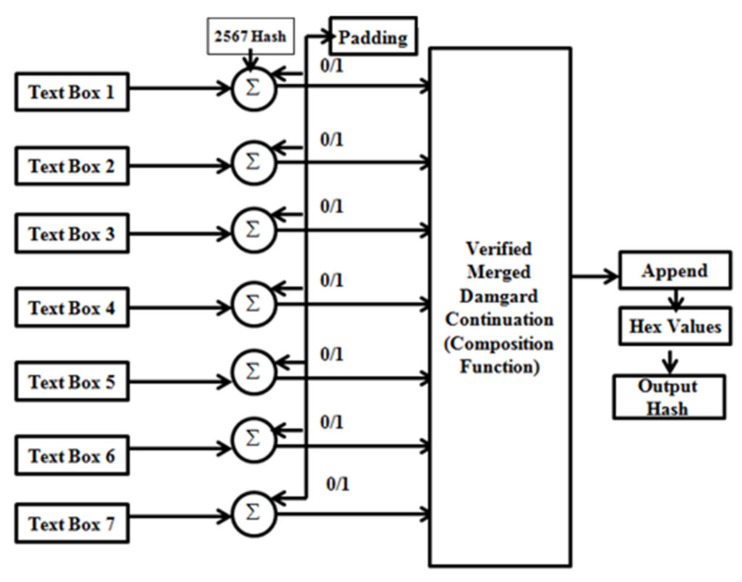
Block diagram of the proposed encryption module.

**Figure 9:**
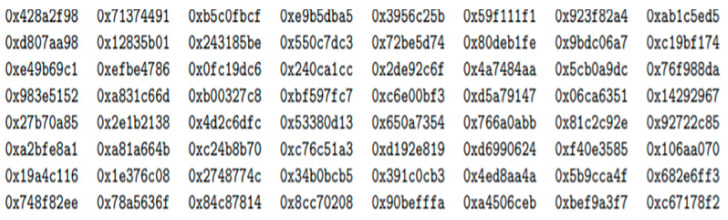
Generated constants that are the keys to the hashing algorithm.

Considering the first textbox function to be T1 in Figure 8, the values from the textbox are first converted into binary format and then get concatenated with the power (2,6) values’ generated constants. The constants are the keys to the hashing algorithm. The constants are obtained as shown in Figure 9.

So, after the concatenation of the textbox 1 values and the constant, we obtain the first character value as:

(3) $$C_{1}=concat\left(T_{1},K\right)$$

Similarly, considering the nth textbox as:

(4) $$C_{n}=concat\left(T_{n},K\right)$$

We then have the input mixture ‘M’ of the hex constants and the binary form of the character.

(5) $$M=\sum_{i=0}^{n}concat\left(T_{i},K\right)$$

Now, there are possibilities of not obtaining a 512-bit as output. Instead, we perform the padding operation of the output by putting 0 s and 1 s to obtain a 512-bit output. After that, we initiate the appending with the compression function. The compression function is the heart of the hash function. In this work, the first hashing input gets concatenated with the compression function. The output gets separated into small blocks of 8-bit data and undergoes repeated padding and compression until the message gets divided into four 4-bit blocks. The compression function used here is the verified Merkle–Damgard construction hashing function [38]. The MD hashing function is given by,

(6) $$MeDa:\left\{0,1\right\}*\to \left\{0,1\right\}n$$

So, we obtain the output of the compression function as:

(7) $$MeDa\left(M\right)=f*\left(padding\left(M\right),public\;value\right)$$

The public value refers to any character string. Finally, we obtain the 256-bit digest and this secured algorithm makes the process irreversible. The compression function satisfies the two important objectives of depressing the size of the digest and avoiding collisions in the hash.

## 3. Results and Discussion

**Figure 10:**
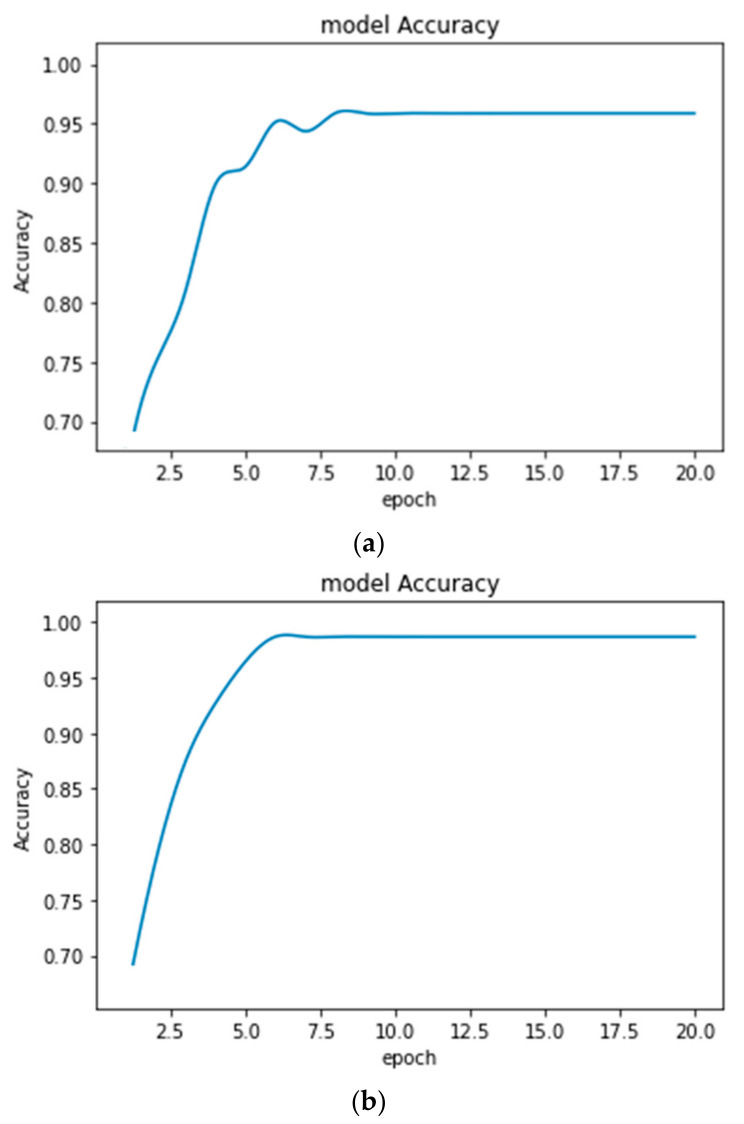
Accuracy plots obtained for Brain MRI classification (**a**) with an earlier form of CE (Accuracy = 95.48%) and (**b**) with improved CE (Accuracy = 98.51%).

**Figure 11:**
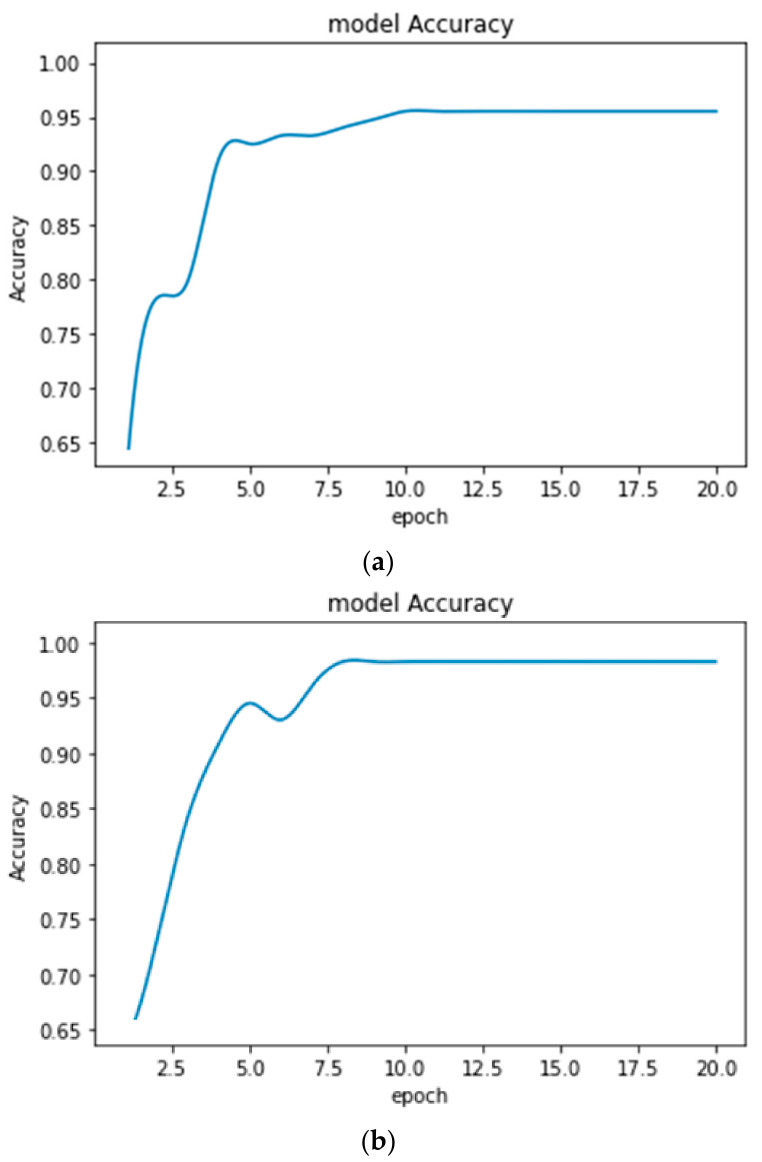
Accuracy plots obtained for EEG NSC-ND data classification (**a**) with earlier form of CE (Accuracy = 95.01%) and (**b**) with improved CE (Accuracy = 98.13%).

The performance of the proposed disease diagnosis models with improved CE increased in comparison to that of the earlier form of CE. The accuracy plots obtained from the proposed models after 20 epochs for MRI and EEG classification are shown in Figure 10 and Figure 11, respectively.

The results obtained in both the conditions for brain tumor detection and seizure detection are given in Table 2 and Table 3, respectively.

From the above Figures showing the accuracy, it is observed that the accuracy plots are smooth enough while using the improved CE algorithm. It indicates the improved training process. The classification models are used for the automatic detection and monitoring of various diseases. The transfer-learning algorithm is adopted so that it can perform well whenever the types of data are changed from patient to patient. The health condition of the patient will provide a further decision.

The hashing algorithm along with the compression function will prevent the attackers from accessing the data as the process will be computationally intense to revert. The algorithm is also the second-preimage-resistive. If the attacker already has obtained the input from the hash and tries to insert a legitimate value in place of the same input, it will not work. It will not be possible to find a different input for the same hash. The hash is free from collisions as the attacker cannot find two different or duplicate values from the hash. The possibility of obtaining two different inputs or duplicative inputs is absent. The algorithm undergoes intensive computation of a mixture of SHA-256 and Merkle–Damgard function. The algorithm is as follows:

1. Preprocessing of inputs:
  - Generate the SHA-2 constant K.
  - Feed the input message from textboxes.
2. Convert the same into binary form.
3. Take the concatenation of the values from textboxes and constant K.
4. Check if padding is required:
  Case 1: If a 512-bit value is generated, then no padding is required.
  Case 2: If 512-bit is not generated, then pad with the 0 s and 1 s to generate the same.
5. Feed the same output into the MD construction compression function:
  - Values get concatenated to the hashes of the compression function.
  - Append the values.
  - Repetitive compression of values.
6. Append the values of the hashing output
7. Result obtained.

**Figure 12:**
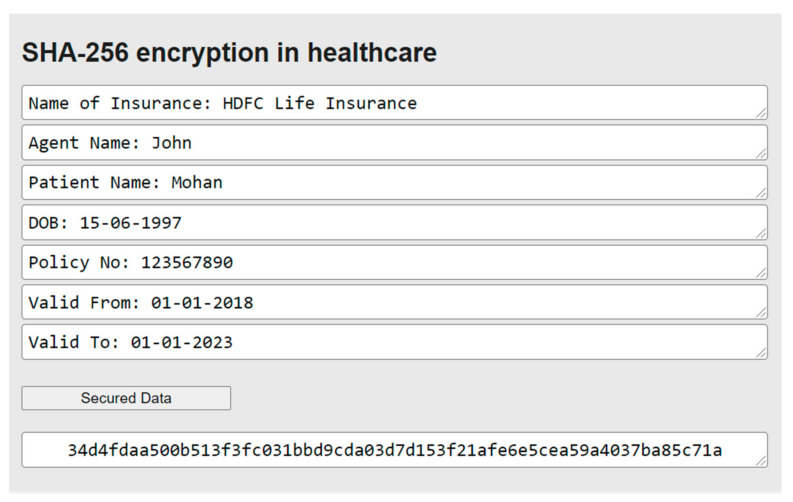
Implementation of the SHA-256 algorithm.

In Figure 12, we can see the implementation of the SHA-256 algorithm in the healthcare web application. There are seven textboxes from which values are taken as input including the insurance details of the patient. In this case, the value of one textbox is first converted into binary format and is concatenated with the hashing constant; similarly, for the other six cases after the concatenation, they are appended and fed to the compression function which then provides the final digest. After entering the inputs in the textboxes, the whole details are encrypted in the form of a 256-bit hash value which is only available for the confidential use of the healthcare system.

**Figure 13:**
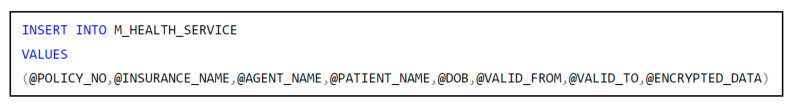
The insert scripts of the data

After the encryption, the data are then pushed to the database for storage and management through the gateway. Microsoft SQL Server is used for database management. The database consists of all the parameters as columns in it. The data from the web application are connected through a gateway to the database. In the database, there is a stored procedure that gets triggered once the data from the web application reach the database server. The stored procedure contains the insert scripts of the data as shown in Figure 13.

**Figure 14:**
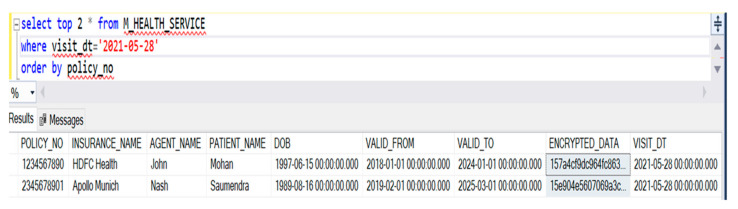
An instance referred to where the admin wants to access the top 2 patient insurance data.

Finally, for accessing the data, we give the secured authentication in the SQL Server. There is one such instance referred to where the admin wants to access the top two patient insurance data for the present date in Figure 14.

This method will be helpful in the current COVID-19 pandemic where people suffering from the virus will be able to claim the insurance with the help of such an e-healthcare system instead of claiming it offline or through insurance cards.

## 4. Conclusions

The smart and secure medical management system will help medical personnel as well as patients in benefitting from a smooth medical service. For this, the authors in this paper attempted to introduce a smart and protected hospital management system. A deep-machine-learning approach with improved cross-entropy is also proposed for automatic disease detection in a short period. This module helps in providing the suggestion of whether a patient should be admitted to the hospital or not. This decision-providing strategy is not yet implemented in *state-of-the-art* methods. A smart and secured health insurance management system is proposed for providing an accurate insurance facility to the patient. For secured insurance data processing, a modified cryptographic approach of SHA-256 is utilized in this work, as it encodes 256 bits into 64 hexadecimal characters providing the highest security. From the result, it can be observed that the proposed approach can be a supportive tool for effective healthcare service. Furthermore, the diagnosis results can also be encrypted using the hashing algorithm.

## Figures and Tables

**Table 1:** Description of the patient registration module.

Input	Description
Name	The user inputs the name of the patient who needs the treatment.
Age	Age of the patient in years.
Address	Address of the patient.
Mobile number	Mobile number of the patient for communication.
Department referred	The patient is referred to the respective department based on the symptoms.
Gender	Gender of the patient (Male/Female/Other)

**Table 2:** Performance obtained on the brain MRI data.

Dataset	Accuracy (%)	
	With the Earlier Form of CE	With Improved CE
Brain MRI	95.48	98.51

**Table 3:** Performance obtained on the EEG data.

Dataset	Accuracy (%)	
	With the Earlier Form of CE	With Improved CE
NSC-ND dataset	95.16	96.60
UoB dataset	95.01	98.13

## Data Availability

The data presented in this study are available on request from the corresponding author.

## References

[1] Mahmud R., Koch F.L., Buyya R. (2018). Cloud-fog interoperability in IoT-enabled healthcare solutions. ACM Int. Conf. Proceeding Ser..

[2] Dorj U.O., Lee M., Choi J.Y., Lee Y.K., Jeong G. (2017). The Intelligent Healthcare Data Management System Using Nanosensors. J. Sens..

[3] Kumar N.M., Mallick P.K. (2018). Blockchain technology for security issues and challenges in IoT. Procedia Comput. Sci..

[4] Matloob I., Khan S.A., Rahman H.U. (2020). Sequence Mining and Prediction-Based Healthcare Fraud Detection Methodology. IEEE Access.

[5] Canlas R.D. (2009). Data mining in healthcare: Data Mining in Healthcare: Current Applications and Issues. Master’s Thesis.

[6] Mohanty M.N., Mohapatra S.K., Pradhan B.B. Multi-Agent Approach based Blood Bank Management System for Emergency Patients. Proceedings of the 2019 Annual Meeting on Management Engineering.

[7] Sarangi L., Mohanty M.N., Patnaik S. (2016). Design of ANFIS based e-health care system for cardio vascular disease detection. Proceedings of the International Conference on Intelligent and Interactive Systems and Applications.

[8] Moosavi S.R., Gia T.N., Nigussie E., Rahmani A.M., Virtanen S., Tenhunen H., Isoaho J. (2016). End-to-end security scheme for mobility enabled healthcare Internet of Things. Futur. Gener. Comput. Syst..

[9] Manikandan R., Patan R., Gandomi A.H., Sivanesan P., Kalyanaraman H. (2020). Hash polynomial two factor decision tree using IoT for smart health care scheduling. Expert Syst. Appl..

[10] Gahlot S., Reddy S.R.N., Kumar D. (2019). Review of smart health monitoring approaches with survey analysis and proposed framework. IEEE Internet Things J..

[11] Saha R., Kumar G., Rai M.K., Thomas R., Lim S.J. (2019). Privacy ensured e-Healthcare for fog-enhanced IoT based applications. IEEE Access.

[12] Ali A., Pasha M.F., Ali J., Fang O.H., Masud M., Jurcut A.D., Alzain M.A. (2022). Deep Learning Based Homomorphic Secure Search-Able Encryption for Keyword Search in Blockchain Healthcare System: A Novel Approach to Cryptography. Sensors.

[13] Garrido A., López L.J.R., Álvarez N.B. (2021). A simulation-based AHP approach to analyze the scalability of EHR systems using blockchain technology in healthcare institutions. Inform. Med. Unlocked.

[14] Saberi M.A., Adda M., Mcheick H. (2022). Break-Glass Conceptual Model for Distributed EHR management system based on Blockchain, IPFS and ABAC. Procedia Comput. Sci..

[15] Fatokun T., Nag A., Sharma S. (2021). Towards a blockchain assisted patient owned system for electronic health records. Electronics.

[16] Mani V., Manickam P., Alotaibi Y., Alghamdi S., Khalaf O.I. (2021). Hyperledger healthchain: Patient-centric IPFS-based storage of health records. Electronics.

[17] Ahene E., Walker J., Gyening R.M.O.M., Abdul-Salaam G., Hayfron-Acquah J.B. (2022). Heterogeneous signcryption with proxy re-encryption and its application in EHR systems. Telecommun. Syst..

[18] Paliwal G., Bunglowala A., Kanthed P. (2022). An architectural design study of electronic healthcare record systems with associated context parameters on MIMIC III. Health Technol..

[19] Ali A., Almaiah M.A., Hajjej F., Pasha M.F., Fang O.H., Khan R., Zakarya M. (2022). An Industrial IoT-Based Blockchain-Enabled Secure Searchable Encryption Approach for Healthcare Systems Using Neural Network. Sensors.

[20] Ali A., Rahim H.A., Ali J., Pasha M.F., Masud M., Rehman A.U., Baz M. (2021). A Novel Secure Blockchain Framework for Accessing Electronic Health Records Using Multiple Certificate Authority. Appl. Sci..

[21] Martsenyuk V., Povoroznyuk V., Semenets A., Martynyuk L. (2019). On an approach of the solution of machine learning problems integrated with data from the open-source system of electronic medical records: Application for fractures prediction. Proceedings of the International Conference on Artificial Intelligence and Soft Computing;.

[22] Huang Z., Du X., Chen L., Li Y., Liu M., Chou Y., Jin L. (2020). Convolutional Neural Network Based on Complex Networks for Brain Tumor Image Classification With a Modified Activation Function. IEEE Access.

[23] Ker J., Singh S.P., Bai Y., Rao J., Lim T., Wang L. (2019). Image thresholding improves 3-dimensional convolutional neural network diagnosis of different acute brain hemorrhages on computed tomography scans. Sensors.

[24] Ker J., Bai Y., Lee H.Y., Rao J., Wang L. (2019). Automated brain histology classification using machine learning. J. Clin. Neurosci..

[25] Ertosun M.G., Rubin D.L. (2015). Automated grading of gliomas using deep learning in digital pathology images: A modular approach with ensemble of convolutional neural networks. AMIA Annu. Symp. Proc..

[26] Ge C., Gu I.Y.H., Jakola A.S., Yang J. (2020). Enlarged training dataset by pairwise GANs for molecular-based brain tumor classification. IEEE Access.

[27] Huda S., Yearwood J., Jelinek H.F., Hassan M.M., Fortino G., Buckland M. (2016). A hybrid feature selection with ensemble classification for imbalanced healthcare data: A case study for brain tumor diagnosis. IEEE Access.

[28] Naga Srinivasu P., Srinivasa Rao T., Dicu A.M., Mnerie C.A., Olariu I. (2020). A comparative review of optimisation techniques in segmentation of brain MR images. J. Intell. Fuzzy Syst..

[29] Srinivasu P.N., Rao T.S., Balas V.E. (2020). Volumetric Estimation of the Damaged Area in the Human Brain from 2D MR Image. Int. J. Inf. Syst. Modeling Des..

[30] Das A., Mohapatra S.K., Mohanty M.N. (2022). Brain Image Classification Using Optimized Extreme Gradient Boosting Ensemble Classifier. Proceedings of the Biologically Inspired Techniques in Many Criteria Decision Making (BITMDM);.

[31] Das A., Mohapatra S.K., Mohanty M.N. (2022). Design of deep ensemble classifier with fuzzy decision method for biomedical image classification. Appl. Soft Comput..

[32] Panda S., Das A., Mishra S., Mohanty M.N. (2021). Epileptic seizure detection using deep ensemble network with empirical wavelet transform. Meas. Sci. Rev..

[33] Chakrabarty N. (2019). Brain MRI Images for Brain Tumor Detection. https://www.kaggle.com/navoneel/brain-mri-images-for-brain-tumor-detection.

[34] Andrzejak R.G., Lehnertz K., Mormann F., Rieke C., David P., Elger C. (2001). Indications of nonlinear deterministic and finite-dimensional structures in time series of brain electrical activity: Dependence on recording region and brain state. Phys. Rev. E.

[35] Gandhi T.K., Chakraborty P., Roy G.G., Panigrahi B.K. (2012). Discrete harmony search based expert model for epileptic seizure detection in electroencephalography. Expert Syst. Appl..

[36] Backes M., Barthe G., Berg M., Grégoire B., Kunz C., Skoruppa M., Béguelin S.Z. (2012). Verified security of merkle-damgård. 2012 IEEE 25th Computer Security Foundations Symposium.

[37] Nidhya R., Shanthi S., Kumar M. (2021). A novel encryption design for wireless body area network in remote healthcare system using enhanced RSA algorithm. Intelligent System Design.

[38] Kumar K.K., Ramaraj E., Srikanth B., Rao A.S., Prasad P.B.V.N. Role of MD5 Message-Digest Algorithm for Providing Security to Low-Power Devices. Proceedings of the 2022 6th International Conference on Intelligent Computing and Control Systems (ICICCS).

